# Supplementary material for: Widening East-West inequality in life expectancy in Europe during the COVID-19 pandemic: An international comparative study
Source: PLoS One. 2026 Feb 27;21(2):e0344003. doi: 10.1371/journal.pone.0344003 (PMC12948044; doi:10.1371/journal.pone.0344003)
Supplement: S1 Appendix — (PDF) [file pone.0344003.s001.pdf]

S1 Appendix. Mortality data

For calculations of the annual life expectancy in 2000-2023 and life expectancy losses in 2020-21, we used country-specific death counts and population exposures from the HMD[1] core that allowed us to compute sex- and age-specific (0, 1-4, 5-9, …, 100+) death rates for every year from 2000 to 2023. For the most recent years in which the final annual data were not yet published for some countries in the HMD core, these death rates were computed from weekly age-specific mortality data from the Input section of the Short-Term Mortality Fluctuations series (STMF)[2].

STMF series was also used as a primary source for assessing weekly series of crude death rates (CDRs) from 2000 to 2021 and excess crude death rates (EDRs) in 2020-21.

---

[1] Barbieri M, Wilmoth JR, Shkolnikov VM, et al. Data Resource Profile: The Human Mortality Database (HMD). Int J Epidemiol. 2015;44(5):1549-56. doi:10.1093/ije/dyv105

[2] Jdanov DA, Galarza AA, Shkolnikov VM, et al. The short-term mortality fluctuation data series, monitoring mortality shocks across time and space. Sci Data. 2021;8(1):235. doi:10.1038/s41597-021-01019-1
