# Supplementary material for: Widening East-West inequality in life expectancy in Europe during the COVID-19 pandemic: An international comparative study
Source: PLoS One. 2026 Feb 27;21(2):e0344003. doi: 10.1371/journal.pone.0344003 (PMC12948044; doi:10.1371/journal.pone.0344003)
Supplement: S2 Appendix — (PDF) [file pone.0344003.s002.pdf]

## S2 Appendix. Calculations from weekly mortality data: the weekly death rates, the timing of the earliest major peaks, and the total excess death rate in 2020-21

The baseline (expected) CDRs by week of 2020-21 were extrapolated from weekly CDRs in 2005-2019 using a variant of the Serfling model that included a quadratic annual trend and two sin-cosine harmonics for seasonal fluctuations[1].

$$CDR_{y,i} = a + by + dy^2 + s_1 \sin\left(\frac{2\pi i}{52}\right) + c_1 \cos\left(\frac{2\pi i}{52}\right) + s_2 \sin\left(\frac{2\pi i}{26}\right) + c_2 \cos\left(\frac{2\pi i}{26}\right) + \varepsilon_{y,i}, (1)$$

with calendar year $y$, week $i$, parameters $a$, $b$, $s_1$, $c_1$, $s_2$, $c_2$, and error term epsilon.

For each country, we defined the earliest major peak of excess mortality as the first local maximum of weekly EDRs exceeding the 95% percentile of weekly EDRs over 2020-2021. In Russia and Estonia, we defined the earliest major peak slightly differently. Due to the very large size of Russia, the timing of the pandemic substantially differed between regions of the country. Consequently, the distribution of weekly EDRs was considerably more dispersed than corresponding distributions in other countries. To address this peculiarity, we used a 90% threshold instead of 95%. In Estonia, the first EDR peak exceeding the 95% threshold took place during the 25th week (June 21-27) of 2021. However, this peak was caused by a heat wave[2]. Therefore, we took the next highest mortality maximum (week 44) as the first major EDR peak in Estonia.

The total EDR values for the groups East and West were computed as averages of country-specific life expectancy losses.

---

[1] Thompson WW, Weintraub E, Dhankhar P, et al. Estimates of US influenza-associated deaths made using four different methods. Influenza Other Respir Viruses. 2009;3(1):37-49. doi:10.1111/j.1750-2659.2009.00073.x;

Simonsen L, Reichert TA, Viboud C, Blackwelder WC, Taylor RJ, Miller MA. Impact of Influenza Vaccination on Seasonal Mortality in the US Elderly Population. Archives of Internal Medicine. 2005;165(3):265-72. doi:10.1001/archinte.165.3.265

[2] 2021 Weather History in Tallinn. 2021. https://weatherspark.com/h/y/91604/2021/Historical-Weather-during-2021-in-Tallinn-Estonia.
