# Supplementary material for: Widening East-West inequality in life expectancy in Europe during the COVID-19 pandemic: An international comparative study
Source: PLoS One. 2026 Feb 27;21(2):e0344003. doi: 10.1371/journal.pone.0344003 (PMC12948044; doi:10.1371/journal.pone.0344003)
Supplement: S3 Appendix — (PDF) [file pone.0344003.s003.pdf]

S3 Appendix. Calculations from weekly mortality data: statistical associations between the excess death rates in March-April 2020 and the flight connectivity across countries in February 2020

Whether a country had a major excess mortality peak in March-April 2020 reflects the number of cases in each country immediately prior to the introduction of wide-ranging measures to reduce transmission within the population. If the number was low, then the sharp reduction in person-to-person mixing that resulted from lock-down measures might have been sufficient to prevent the establishment of a self-sustaining, exponential growth in cases. Within Europe, by February 2020, most of these initial cases would have been imported from other European countries, such as Italy which experienced the earliest peak in the region, as direct flights from China to Europe had already been severely reduced.

The country-specific excess death rates in April-May 2020 were computed by averaging weekly EDRs over weeks 10 to 18. The flight connectivity was measured by the average daily number of flights arriving in each country from other European countries during two weeks around the middle of February (weeks 10 to 18) 2020. This information was obtained by extracting flight records from the air traffic control data set by the *OpenSky* Network[1] and their tabulation by country and week of 2020 (Supplementary Table S2).

Preliminary analysis showed that both the excess death rate and the arriving flight variables have highly skewed distributions that could not be treated by a log transformation. We therefore transformed the two originally continuous variables into rankings. The statistical association between the two resulting ordinal variables was measured by Spearman's *rho* and Kendall's *tau* coefficients.

[1] Strohmeier M. OpenSky COVID-19 Flight Dataset. https://opensky-network.org/community/blog/item/6-opensky-covid-19-flight-dataset.
