# Supplementary material for: Widening East-West inequality in life expectancy in Europe during the COVID-19 pandemic: An international comparative study
Source: PLoS One. 2026 Feb 27;21(2):e0344003. doi: 10.1371/journal.pone.0344003 (PMC12948044; doi:10.1371/journal.pone.0344003)
Supplement: S4 Appendix — (PDF) [file pone.0344003.s004.pdf]

S4 Appendix. Calculations from annual mortality data by age and sex: the life expectancy losses in 2020-21 and their split by broad age group

Abridged life tables for all 28 populations for years 2000 to 2023 were estimated from the observed age-specific death rates by applying the standard life table technique[1]. The life expectancy at birth series was extracted from these life tables.

The excess mortality estimates may be sensitive to the choice of the mortality index, the length of the reference (fitting) period, and the method for predicting the baseline mortality level[2]. In our analysis, life expectancy at birth is our ultimate mortality measure. Its choice is not in question. Due to the dominance of the well-known Lee-Carter model in forecasting life expectancy, we use this conventional method for determining the baseline values of life expectancy in 2020-21. The length of the reference (fitting) period is the only parameter to be chosen in our study. We used 2005-19 based on the experience of STMF and earlier sensitivity analyses.[3]

Thus, the Lee-Carter model was applied for forecasting the baseline age- and sex-specific death rates and abridged life tables for 2020 and 2021, using 2005-2019 as the reference (fitting) period. Noteworthy, our estimates of the baseline life expectancies and the life expectancy losses were almost the same as those by Schöley et al.[4], who used 2015-19 as the reference period. The life expectancy losses in 2020 and 2021 were computed as the differences between the baseline and the observed life expectancy values in 2020 and 2021. We also estimated 95% confidence limits for these quantities by carrying out 2000 bootstrap simulations nested on prediction errors of the age-specific death rates.

From the baseline and the observed life tables, we extracted the baseline and the observed life expectancies at ages 0 and 65, interval life expectancies between exact ages 0 and 65, and the probabilities of survival to age 65. Following the counterfactual replacement algorithm[5], the contribution of ages 0-64 to the overall life expectancy loss was expressed as:

$$\delta_{0-64} = [(l_0^* e_0^* - l_{65}^* e_{65}^*) - (l_0 e_0 - l_{65} e_{65})] + \frac{1}{2} \cdot [(l_{65}^* - l_{65}) e_{65}^* - (l_{65} - l_{65}^*) e_{65}], \quad (2)$$

[1] Wilmoth JR, Andreev KD, Jdanov DA, Glei D, Riffe T. Methods Protocol for the Human Mortality Database. https://www.mortality.org/File/GetDocument/Public/Docs/MethodsProtocolV6.pdf. 2021.

[2] Nepomuceno MR, Klimkin I, Jdanov DA, Alustiza-Galarza A, Shkolnikov VM. Sensitivity Analysis of Excess Mortality due to the COVID-19 Pandemic. Popul Dev Rev. 2022;48(2):279-302. doi:10.1111/padr.12475

[3] Nepomuceno MR, Klimkin I, Jdanov DA, Alustiza-Galarza A, Shkolnikov VM. Sensitivity Analysis of Excess Mortality due to the COVID-19 Pandemic. Popul Dev Rev. 2022;48(2):279-302. doi:10.1111/padr.12475; Booth H, Hyndman RJ, Tickle L, de Jong P. Lee-Carter mortality forecasting: a multi-country comparison of variants and extensions. Demographic Research. 2006;15:289-310. doi:10.4054/DemRes.2006.15.9

[4] Scholey J, Aburto JM, Kashnitsky I, et al. Life expectancy changes since COVID-19. Nat Hum Behav. 2022. doi:10.1038/s41562-022-01450-3

[5] Andreev EM, Shkolnikov V, Begun A. Algorithm for decomposition of differences between aggregate demographic measures and its application to life expectancies, healthy life expectancies, parity-progression ratios and total fertility rates. Demographic Research. 2002;7(14):499-522. doi:10.4054/DemRes.2002.7.14

where e_0, e_0^* and e_{65}, e_{65}^* are observed/baseline life expectancies at ages 0 and age 65, respectively, in the year 2020 or 2021; l_0, l_0^* and l_{65}, l_{65}^* are observed/baseline life table numbers of survivors to ages 0 and 65, respectively. The two additive terms on the right-hand side of Eq. 2 are equal to: 1) the lifetime loss between exact ages 0 and 65 produced by the observed-baseline mortality differences at ages 0 to 64; 2) the lifetime lost at ages higher or equal to 65 produced by the observed-baseline mortality differences at ages 0 to 64.

The contribution of ages 65+ was estimated as the difference between the overall life expectancy loss δ and the one imposed by younger ages δ_{0-64}:

$$\delta_{65+} = \delta - \delta_{0-64}.$$
