# Supplementary material for: Widening East-West inequality in life expectancy in Europe during the COVID-19 pandemic: An international comparative study
Source: PLoS One. 2026 Feb 27;21(2):e0344003. doi: 10.1371/journal.pone.0344003 (PMC12948044; doi:10.1371/journal.pone.0344003)
Supplement: S5 Appendix — (PDF) [file pone.0344003.s005.pdf]

S5 Appendix. Calculations from annual mortality data by age and sex: contributions of relative mortality excess and baseline mortality to the total East-West differences in the life expectancy losses in 2021

The life expectancy losses in a *target* Eastern population and a *reference* Western population are

$$\delta_E = e^*_{0,E} - e_{0,E} = e_0(\boldsymbol{M}^*_E) - e_0(\boldsymbol{M}_E)\,, \qquad (3a)$$

$$\delta_W = e^*_{0,W} - e_{0,W} = e_0(\boldsymbol{M}^*_W) - e_0(\boldsymbol{M}_W), \qquad (3b)$$

where $\boldsymbol{M}_E, \boldsymbol{M}_W, \boldsymbol{M}^*_E, \boldsymbol{M}^*_W$ are the observed and the baseline vectors of age-specific death rates, and the life expectancy at birth is expressed as a scalar function of these vectors.

The target losses may exceed the reference losses because of the stronger elevation of the observed death rates above the baseline in the East compared to the West and because of the higher level and younger age distribution of the baseline death rates in the East compared to the West. Correspondingly, the East-West difference in the life expectancy losses $\Delta_{E-W} = \delta_E - \delta_W$ is considered as being dependent on A) relative mortality excess (*Change* component); B) different baseline mortality including both its level and its age shape (*Level* component). The East-West difference Δ_{E-W} can be presented as a sum of these two components:

$$\Delta_{E-W} = \Delta_C + \Delta_L. \qquad (4)$$

The calculation formulae for the components Δ_C and Δ_L are given in Shkolnikov et al.[1]. They use a simple counterfactual approach by computing (for example) the hypothetical life expectancy losses in the target population by combining the proportional mortality excess of this population with the baseline death rates of the reference population.

---

[1] Shkolnikov VM, Jdanov D, Leon DA. Decomposition of differences between life expectancy losses or gains: relative change-related and absolute level components. A Research Note 2023.
