# Supplementary material for: Widening East-West inequality in life expectancy in Europe during the COVID-19 pandemic: An international comparative study
Source: PLoS One. 2026 Feb 27;21(2):e0344003. doi: 10.1371/journal.pone.0344003 (PMC12948044; doi:10.1371/journal.pone.0344003)
Supplement: S6 Appendix — (PDF) [file pone.0344003.s006.pdf]

S6 Appendix. Statistical associations between the East-West differences in life expectancy losses in 2021 and explanatory variables across countries

The set of explanatory variables included proxy variables for factors shown in the conceptual diagram (Figure 1 of the Main text):

- *Percentage of fully vaccinated as of September 1, 2021[1];*

This variable reflects the speed of the vaccination campaigns in different countries, which were typically launched between December 2020 and March 2021. It is also highly correlated with vaccination coverage on December 1 and December 31, 2021.

- *Mean Stringency Index over weeks of 2021[2];*

This variable reflects the average exposure of the population to a range of non-pharmaceutical anti-pandemic measures in 2021. Our attempts to construct a more advanced measure of stringency, possibly combining weekly stringency indices with the weekly intensity of registered cases of COVID-19, did not yield a meaningful result.
Therefore, we used the widely known Stringency Index (SI) by the Oxford Government Response Tracker (OxCGRT), allowing one to compare the strictness of government responses to the COVID-19 pandemic. SI is a score based on the averaging of eight indicators coded on the ordinal scale (0-3 or 0-2) depending on the level of government intervention. These are: 1) closings of schools and universities; 2) closings of workplaces requiring work from home; 3) cancelling public events; 4) limits on gatherings; 5) closing of public transportation requiring prohibiting most citizens from using it; 6) stay-at-home requirements; 7) restrictions on internal movement between cities/regions; and 8) restrictions on international travel for foreign travelers.

- *Percentage of people who trust the national government reported in 2020[3];*

Percentage of respondents who answered "a lot" or "some" when asked: "How much do you trust your national government?" Trust is a central component of social capital. Trust in national governments can influence people's level of skepticism about messages, decrees, and recommendations from state authorities, which in turn can influence the practical adherence to protective measures and restrictions at the individual, collective and institutional levels.

- *Percentage of people who trust science reported in 2020[3] ;*

Percentage of respondents who answered "a lot" or "some" to the question: "How much do you trust science? Trust in science may influence people's attitudes toward

---

[1] Mathieu E, Ritchie H, Ortiz-Ospina E, et al. A global database of COVID-19 vaccinations. Nat Hum Behav. 2021;5(7):947-53. doi:10.1038/s41562-021-01122-8.

[2] Hale T, Angrist N, Goldszmidt R, et al. A global panel database of pandemic policies (Oxford COVID-19 Government Response Tracker). Nat Hum Behav. 2021;5(4):529-38. doi:10.1038/s41562-021-01079-8.

[3] Wellcome Trust. Wellcome Global Monitor 2020:Covid-19. https://wellcome.org/reports/wellcome-global-monitor-covid-19/2020. Last accessed 27/8/2022.: Wellcome Trust2020.

vaccination and scientifically approved behaviours recommended for self-protection and protection of others.

- *Regulatory Enforcement Score in 2021*[4].

This index constitutes Factor 6 (the Regulatory Enforcement - RE) of the World Justice Project's Rule of Law Index, used for over 140 countries. It measures the extent to which regulations are effectively and fairly implemented and enforced.
The RE factor is based on both expert judgment and survey data. The factor uses data from the General Population Poll (GPP), a large survey of the general public, and the Qualified Respondents' Questionnaire (QRQ), a survey of legal professionals and experts. The RE factor is calculated by averaging four sub-scores, which reflect the effectiveness of enforcement, the absence of improper influences, the timeliness of implementation, and the consistency and fairness of related procedures. Each of the sub-scores is composed of multiple questions from GPP and QRQ surveys. The individual-level data is further aggregated and normalized to produce comparable country-level scores.
In our study, RE is considered a characteristic of the perceived ability of authorities to implement and enforce the full range of anti-pandemic measures efficiently and quickly.

To justify the applicability of Pearson's correlation coefficients and linear OLS models for linking life expectancy losses to the explanatory variables, we checked the normality of variables' distributions by their visual examination and by executing Shapiro-Wilk and Skewness/Kurtosis tests. We then carried out statistical tests for linearity and heteroscedasticity (Breusch-Pagan/Cook-Weisberg test) of relationships between the life expectancy losses and each explanatory variable.

To obtain normality of the vaccination variables, we trimmed one outlying point for Bulgaria by replacing the original value with the average of the 1st and the 5th percentiles of the distribution of this variable. We have not found any evidence for the non-normality of the residuals. There was also no evidence of non-linearity of the relationships. Finally, we found evidence of heteroscedasticity in the relationships between the male and female life expectancy losses on one side and the regulation enforcement and trust in the national government variables on the other. We addressed it by using robust standard errors in the corresponding regression models.

After completing the tests, we regressed male and female life expectancy losses across countries on vaccination as of the 1st of September 2021, trust in government, trust in science, and regulatory enforcement.

Finally, we examined the East-West difference in male and female life expectancy losses (presented by the East-West dummy) across countries and how this difference attenuates in response to adjustment of the regression model for single explanatory variables

---

[4] WJP Rule of Law Index 2021. https://worldjusticeproject.org/rule-of-law-index/global/2021/ Last accessed 27/08/22 [database on the Internet]2022. Available from: https://worldjusticeproject.org/rule-of-law-index/global/2021/.

and two-variable combinations. The use of the two-variable models was justified through the fit criteria: the root mean squared error (RMSE), and the Bayesian information criterion (BIC).

$$\delta_i = d \cdot EW + \varepsilon_i, \tag{5a}$$

$$\delta_i = d \cdot EW + a \cdot v1_i + \varepsilon_i, \tag{5b}$$

$$\delta_i = d \cdot EW + a \cdot v1_i + b \cdot v2_i + \varepsilon_i\ . \tag{5c}$$

,

where δ_i is male or female life expectancy loss for country *i,* EW is the East-West dummy (=1 for eastern countries, =0 for western countries), $v1_i$ and $v2_i$ are values of two explanatory variables for a country i, and ε_i is the error term for country i. The degree of attenuation is reflected by the reduction of *d* in model (5b) or model (5c) compared to model (5a).
