# Supplementary material for: Widening East-West inequality in life expectancy in Europe during the COVID-19 pandemic: An international comparative study
Source: PLoS One. 2026 Feb 27;21(2):e0344003. doi: 10.1371/journal.pone.0344003 (PMC12948044; doi:10.1371/journal.pone.0344003)
Supplement: S1 Fig — (PDF) [file pone.0344003.s007.pdf]

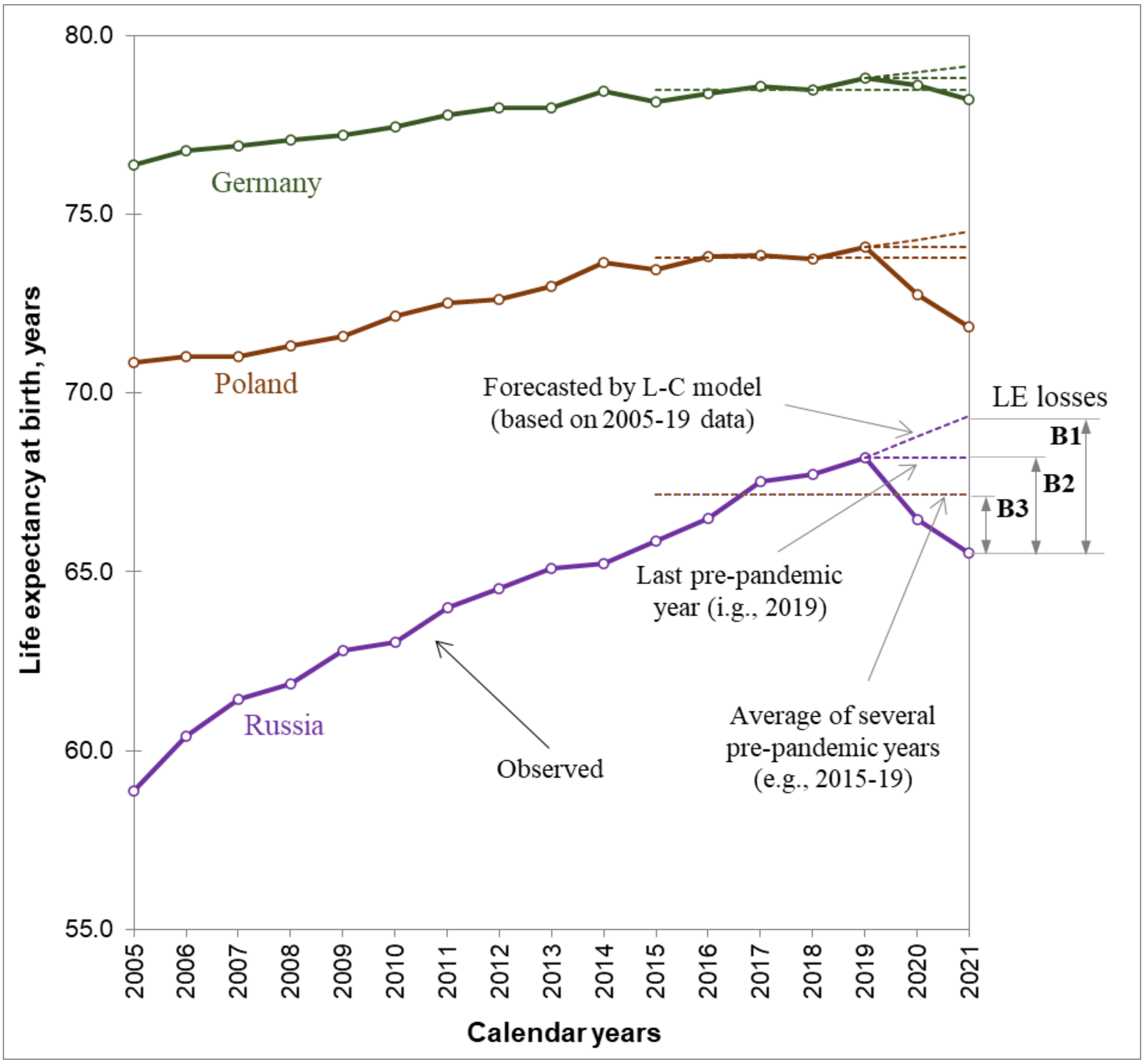

S1 Fig. A graphic presentation of different approaches to estimating baseline mortality and corresponding life expectancy losses in 2020-2021: an example of Russia, Poland, and Germany.

The figure suggests that the usage of baselines corresponding to the average life expectancy in 2015-19 or to the life expectancy in 2019 results in an underestimation of the life expectancy losses in 2021. The degree of underestimation is higher in populations with initially lower levels of life expectancy and its steeper before 2020 such as Russia.

Notation

B1: Baseline 1 - Lee-Carter predicted life expectancy from age-specific death rates in 2005-2019. B2: Baseline 2 - life expectancy in 2019; B3: Baseline 3 - average life expectancy in 2015-19. Use of Baselines 2 and 3 leads to underestimation of life expectancy losses if life expectancy increases with time.

Data shown in this Figure is provided at https://github.com/VMSdemo/East-West-contrast-in-life-expectancy-losses-in-2020-21
