# Supplementary material for: Widening East-West inequality in life expectancy in Europe during the COVID-19 pandemic: An international comparative study
Source: PLoS One. 2026 Feb 27;21(2):e0344003. doi: 10.1371/journal.pone.0344003 (PMC12948044; doi:10.1371/journal.pone.0344003)
Supplement: S2A Fig — (PDF) [file pone.0344003.s008.pdf]

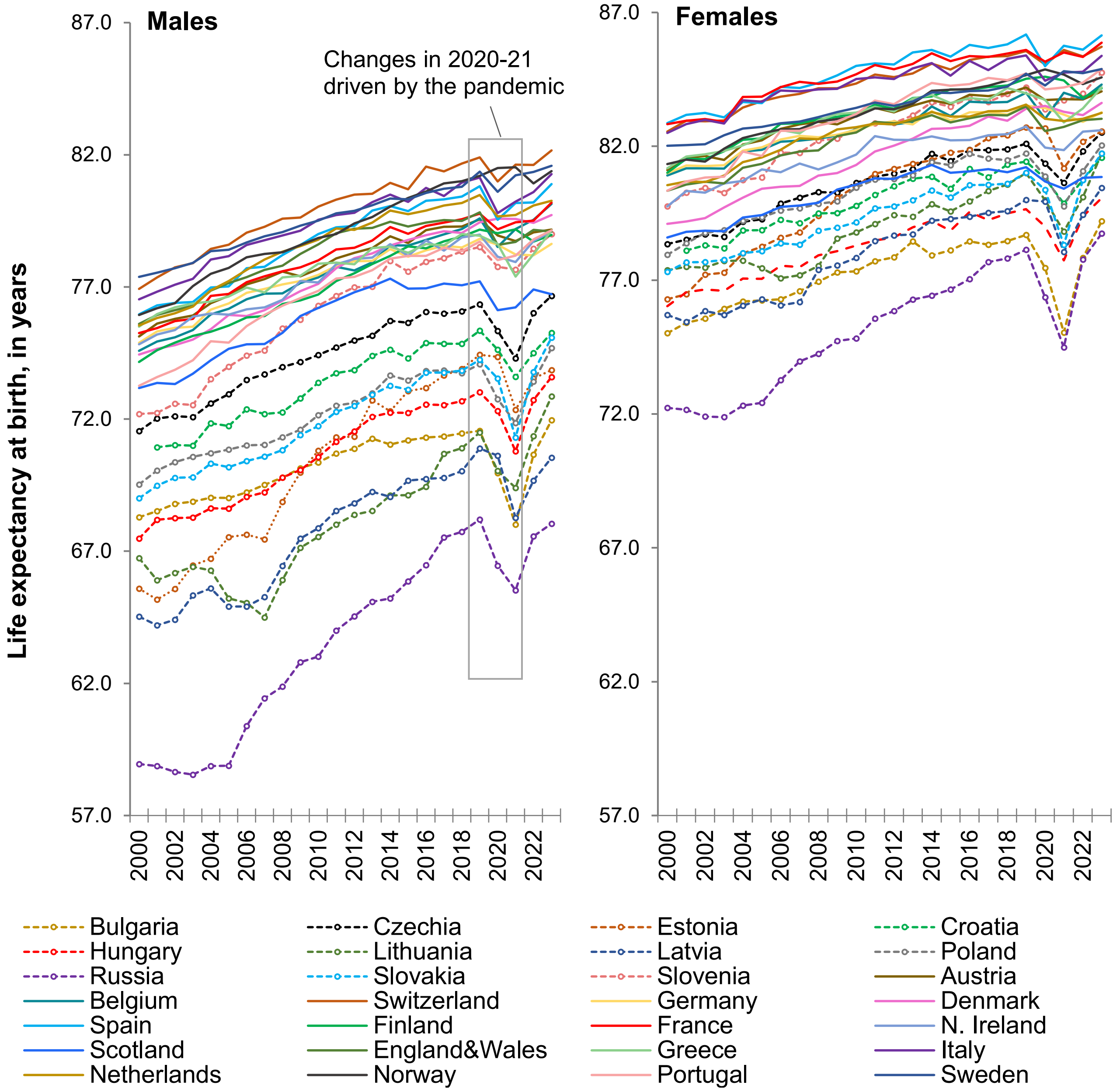

S2A Fig. Trends in life expectancy at birth in 2000-2023, by sex.

The figure shows the increasing life expectancy at birth in Eastern and Western European countries.

Data shown in this Figure is provided at https://github.com/VMSdemo/East-West-contrast-in-life-expectancy-losses-in-2020-21.
