# Supplementary material for: Widening East-West inequality in life expectancy in Europe during the COVID-19 pandemic: An international comparative study
Source: PLoS One. 2026 Feb 27;21(2):e0344003. doi: 10.1371/journal.pone.0344003 (PMC12948044; doi:10.1371/journal.pone.0344003)
Supplement: S2B Fig — (PDF) [file pone.0344003.s009.pdf]

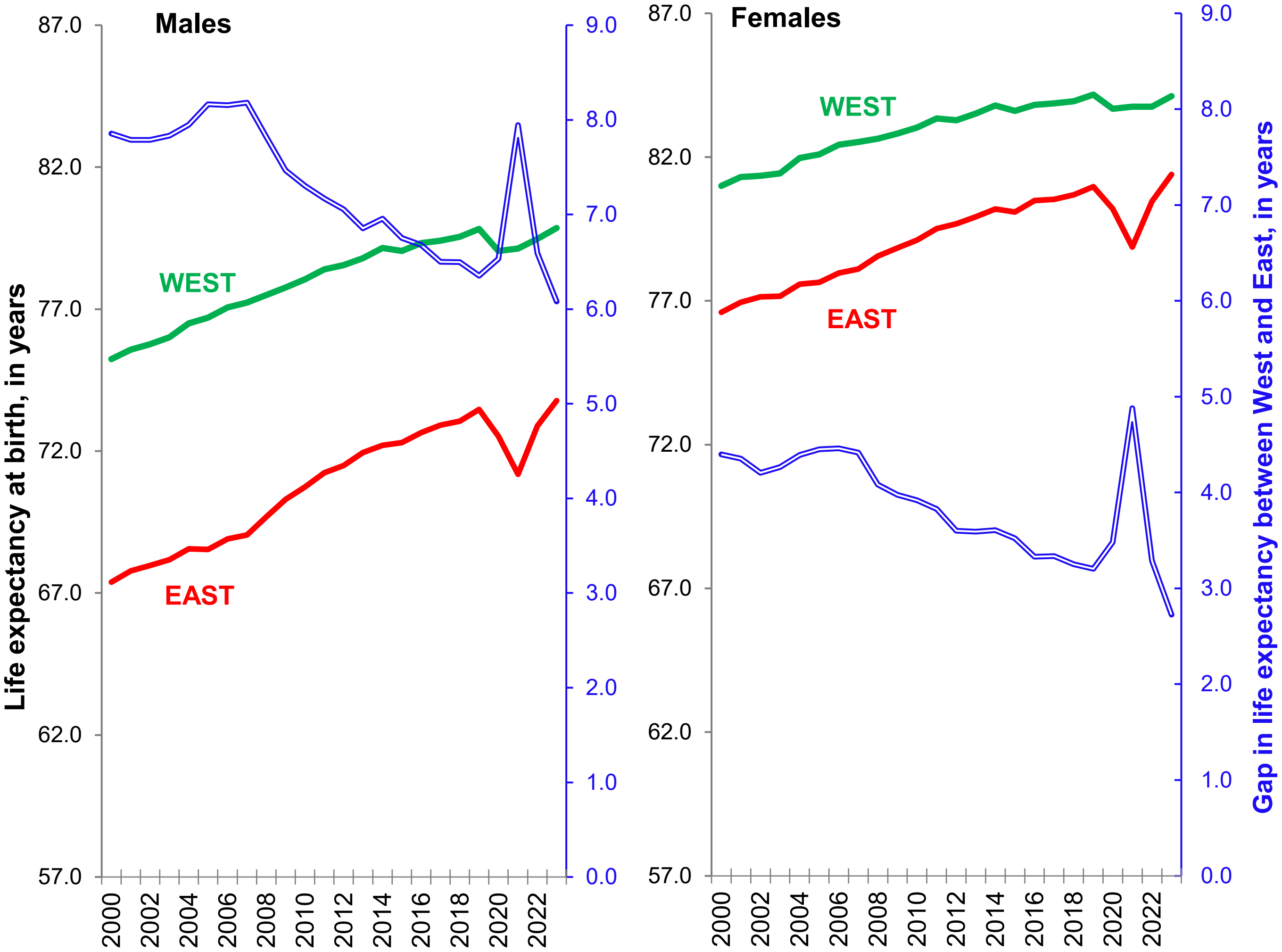

S2B Fig. Trends in life expectancy at birth (2000–2023) and the gap in life expectancy between West and East, by sex.

The figure shows the trends in average life expectancy for East (bold green) and West (bold red) and gap in life expectancy between West and East. The long-term gap in life expectancy between East and West narrowed in the period 2000-2019, due to stronger improvements in the East. The COVID-19 pandemic abruptly reversed the improvement and convergence. After 2021, the pre-pandemic improvement and convergence resumed, but life expectancy in 2023 is mostly below the pre-pandemic trend.
