# Supplementary material for: Widening East-West inequality in life expectancy in Europe during the COVID-19 pandemic: An international comparative study
Source: PLoS One. 2026 Feb 27;21(2):e0344003. doi: 10.1371/journal.pone.0344003 (PMC12948044; doi:10.1371/journal.pone.0344003)
Supplement: S3 Fig — (PDF) [file pone.0344003.s010.pdf]

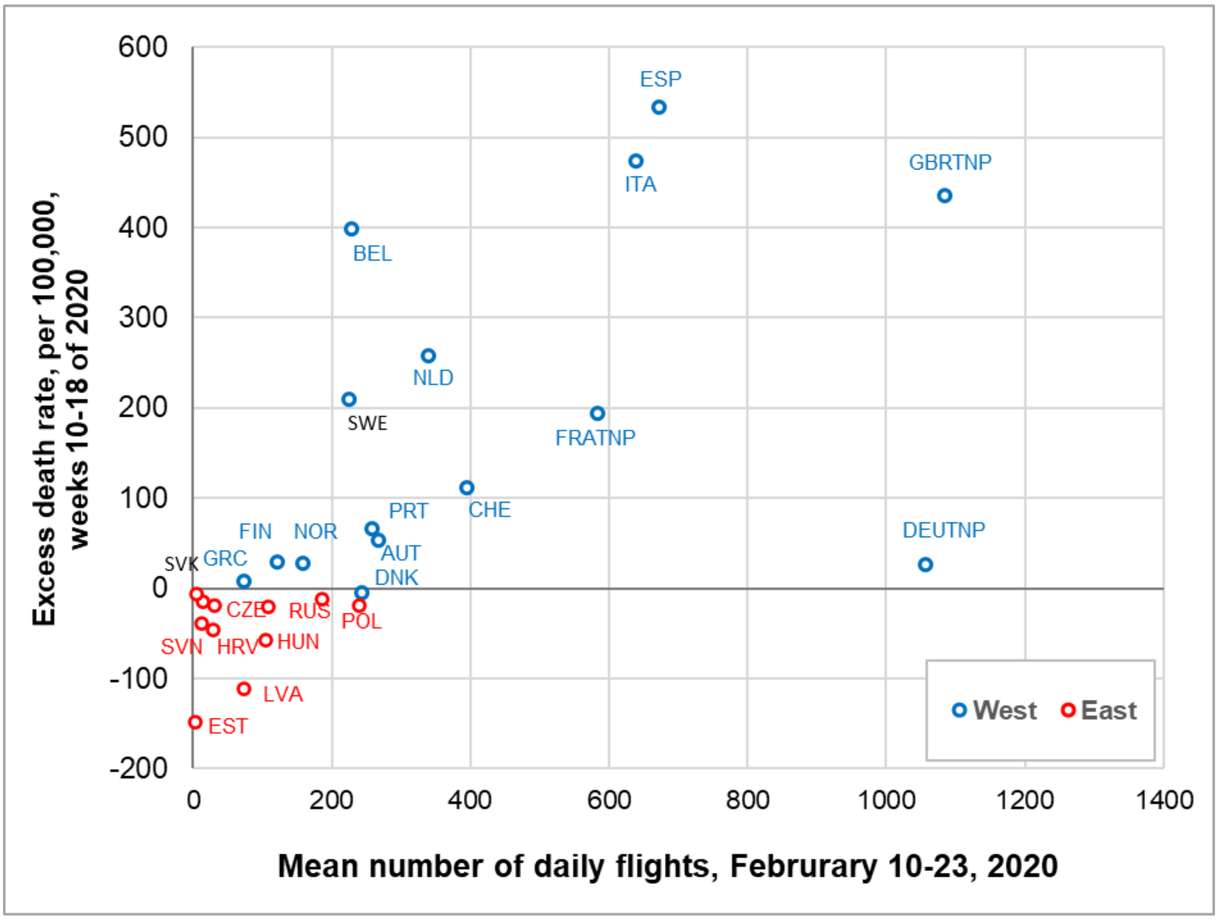

S3 Fig. Correlation between the excess death rate in March-April (weeks 10 to 18), 2020, and the daily number of arriving flights for the period February 10 to 23, 2020.

The Figure shows the much lower connectivity of Eastern European countries compared to those of Western Europe, together with a correlation across all European countries between immediate pre-pandemic connectivity and excess death rates in the first few months of the pandemic.

Country codes and names:
BGR-Bulgaria, CZE-Czechia, EST-Estonia, HRV-Croatia, HUN-Hungary, LTU-Lithuania, LVA-Latvia, POL-Poland, RUS-Russia, SVK-Slovakia, SVN-Slovenia
AUT-Austria, BEL-Belgium, CHE-Switzerland, DEUTNP-Germany, DNK-Denmark, ESP-Spain, FIN-Finland, FRATNP-France, GBRTNP-the UK, GRC-Greece, ITA-Italy, NLD-Netherlands, NOR-Norway, PRT-Portugal, SWE-Sweden

Data shown in this Figure is provided at https://github.com/VMSdemo/East-West-contrast-in-life-expectancy-losses-in-2020-21
