# Supplementary material for: Widening East-West inequality in life expectancy in Europe during the COVID-19 pandemic: An international comparative study
Source: PLoS One. 2026 Feb 27;21(2):e0344003. doi: 10.1371/journal.pone.0344003 (PMC12948044; doi:10.1371/journal.pone.0344003)
Supplement: S4 Fig — (PDF) [file pone.0344003.s011.pdf]

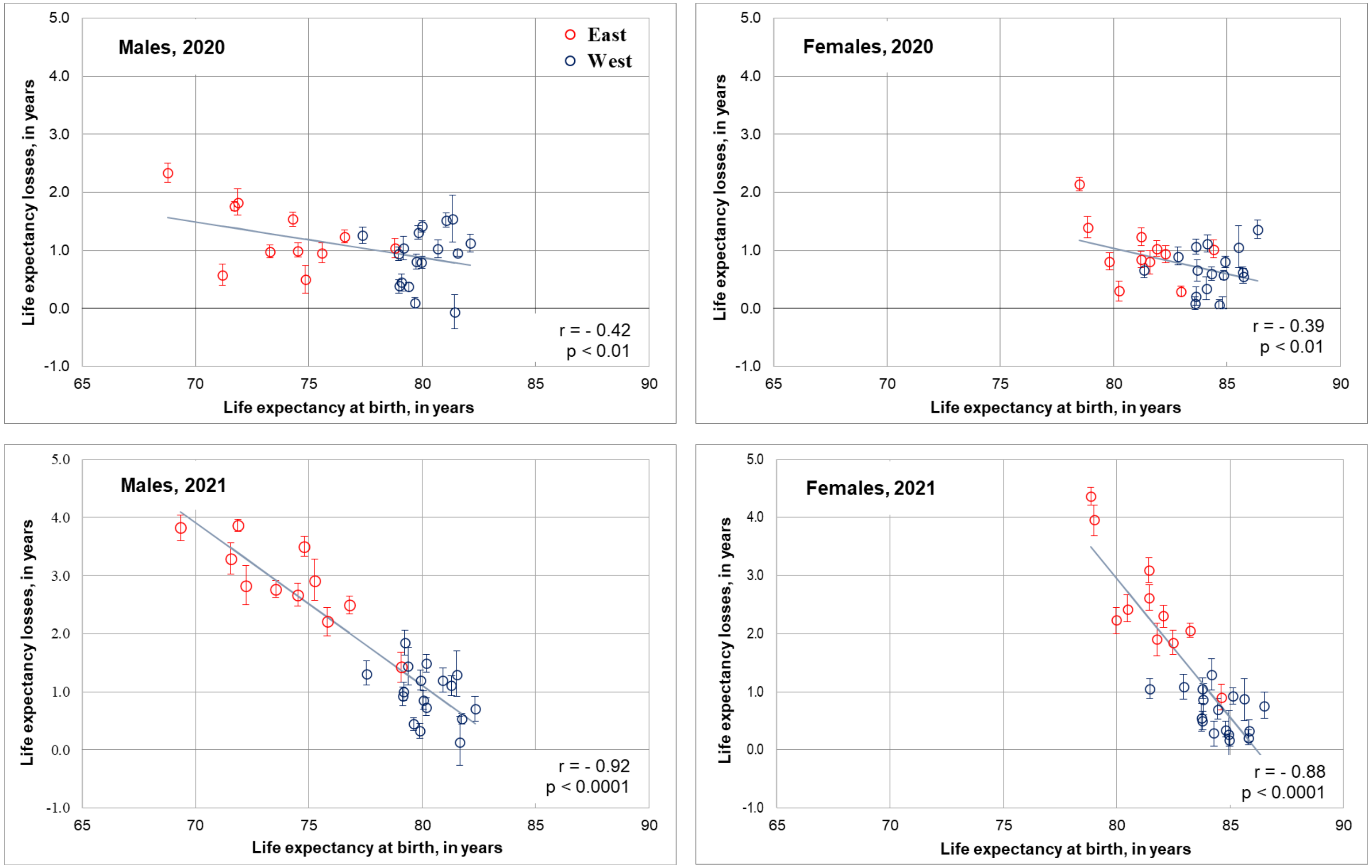

S4 Fig. Associations between life expectancy losses and predicted life expectancies in 2020 and 2021, by sex.

Life expectancy loss shows the difference between observed and expected (in the absence of the pandemic) life expectancies. A pronounced East-West difference in life expectancy losses became apparent in 2021. In this year, the lower level of life expectancy was strongly associated with higher life expectancy loss. Data shown in this Figure is provided at https://github.com/VMSdemo/East-West-contrast-in-life-expectancy-losses-in-2020-21
