# Supplementary material for: Widening East-West inequality in life expectancy in Europe during the COVID-19 pandemic: An international comparative study
Source: PLoS One. 2026 Feb 27;21(2):e0344003. doi: 10.1371/journal.pone.0344003 (PMC12948044; doi:10.1371/journal.pone.0344003)
Supplement: S5 Fig — (PDF) [file pone.0344003.s012.pdf]

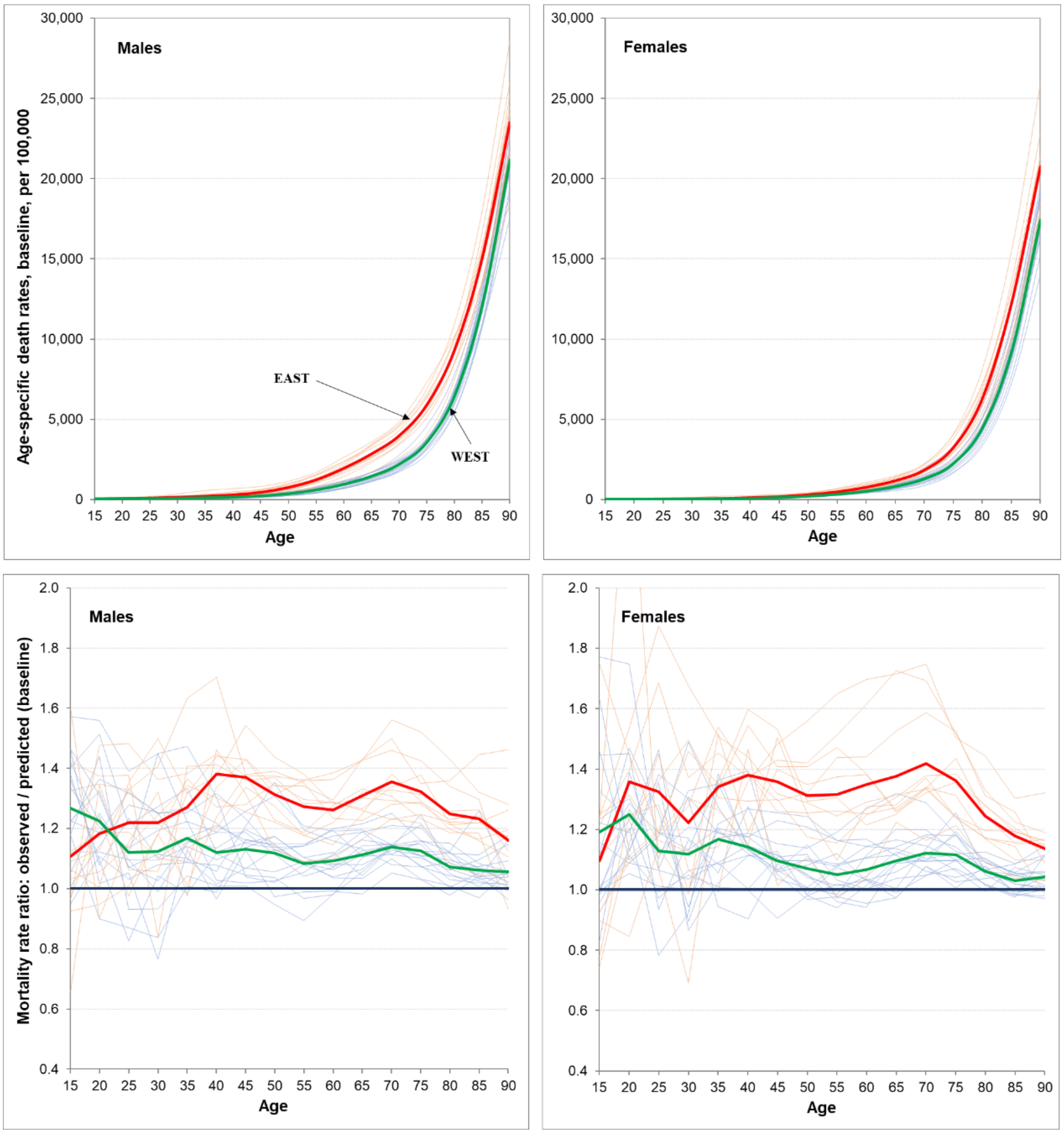

S5 Fig. East-West contrasts in baseline mortality (upper panels) and relative (ratio observed / baseline) mortality excess, by age in 2021.

The upper panels show the baseline age-specific death rates for countries (thin lines) as well as the mean rates for the East and West groups (thick lines). The lower panels show the ratios of the observed to the baseline age-specific death rates. While the East-West divide in the baseline (especially at ages under 70-75) reflects long-term levels of population health, the relative excess reflects the elevation in mortality caused by the pandemic.

In the upper panel, an exponential increase with age in baseline (predicted) death rates in 2021 is seen in both Eastern and Western Europe for both sexes, with substantially higher death rates in the East. The lower panel shows relative excess death rates (observed/baseline rate ratio) in 2021. These fluctuate without systematic increase or decrease between ages 35 and 75 years in both Eastern and Western Europe. However, the extent of the relative excess is much larger in the East than in the West. For males, the excess is about 30%-40% in the East and 10%-15% in the West. For females, the corresponding values are 40%-45% in the East and 5%-15% in the West.

Data shown in this Figure is provided at https://github.com/VMSdemo/East-West-contrast-in-life-expectancy-losses-in-2020-21 .
