# Supplementary material for: Widening East-West inequality in life expectancy in Europe during the COVID-19 pandemic: An international comparative study
Source: PLoS One. 2026 Feb 27;21(2):e0344003. doi: 10.1371/journal.pone.0344003 (PMC12948044; doi:10.1371/journal.pone.0344003)
Supplement: S6 Fig — (PDF) [file pone.0344003.s013.pdf]

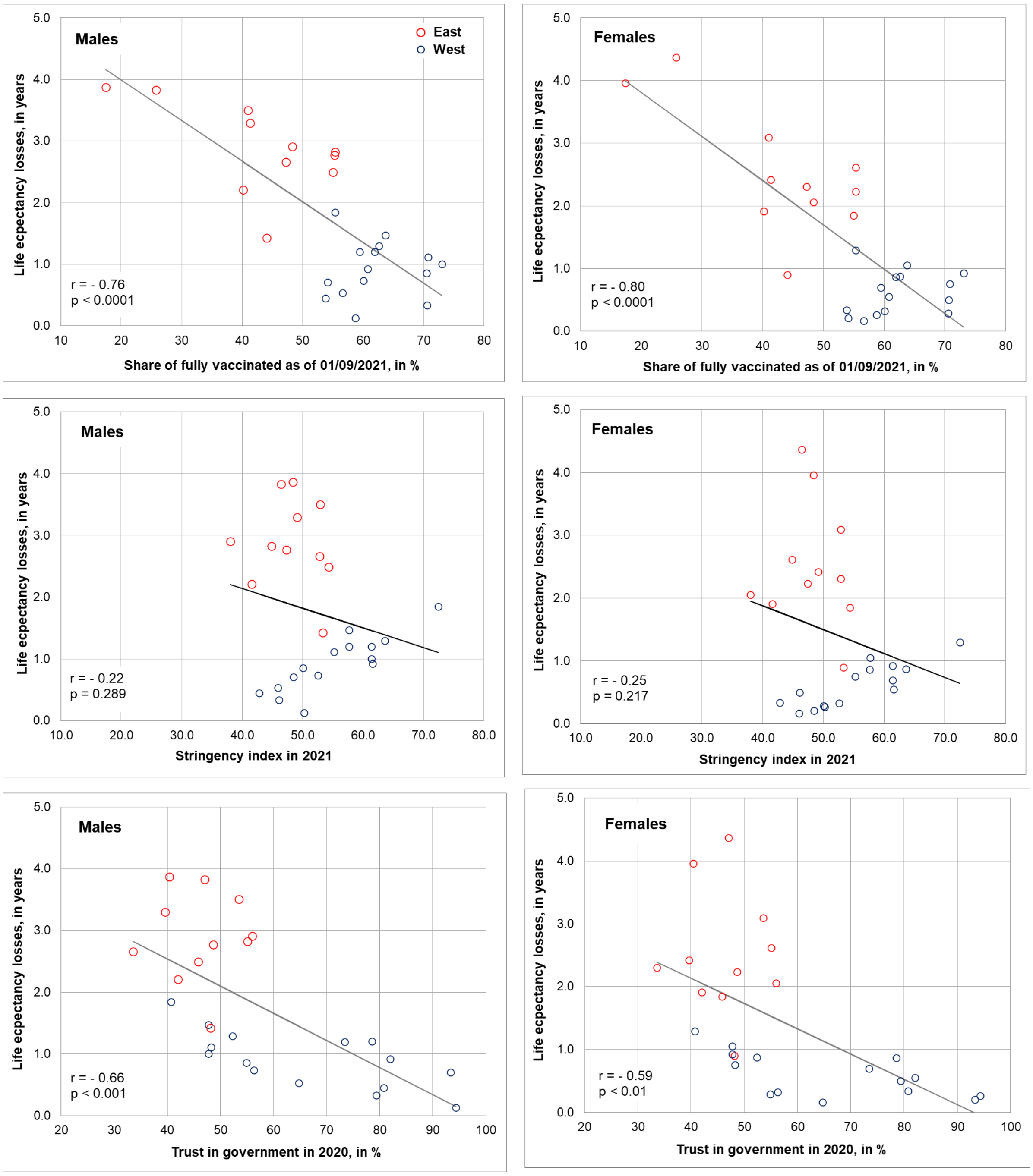

S6 Fig. Associations between sex-specific life expectancy losses in 2021 with single explanatory factors across countries.

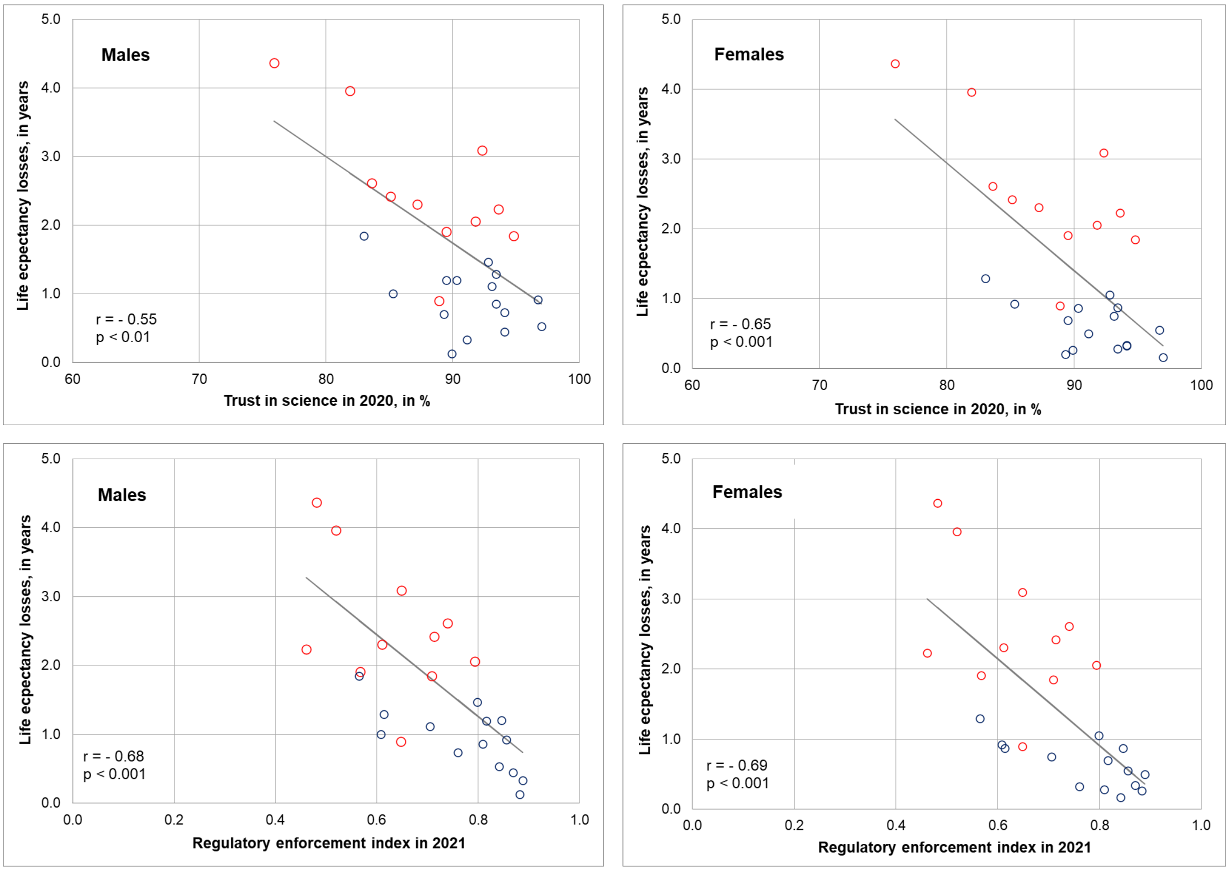

S6 Fig (cont.). Associations between sex-specific life expectancy losses in 2021 with single explanatory factors across countries.

The figure shows OLS regression lines and Pearson correlation coefficients. Value of the Pearson's *r* are shown in the panels. Life expectancy losses are negatively correlated with vaccination, trust, and regulatory enforcement variables across countries. No statistically significant correlations were found for the stringency index.

Data shown in this Figure is provided at [https://github.com/VMSdemo/East-West-contrast-in-life-expectancy-losses-in-2020-21.](https://github.com/VMSdemo/East-West-contrast-in-life-expectancy-losses-in-2020-21)
