# Supplementary material for: Widening East-West inequality in life expectancy in Europe during the COVID-19 pandemic: An international comparative study
Source: PLoS One. 2026 Feb 27;21(2):e0344003. doi: 10.1371/journal.pone.0344003 (PMC12948044; doi:10.1371/journal.pone.0344003)
Supplement: S7 Fig — (PDF) [file pone.0344003.s014.pdf]

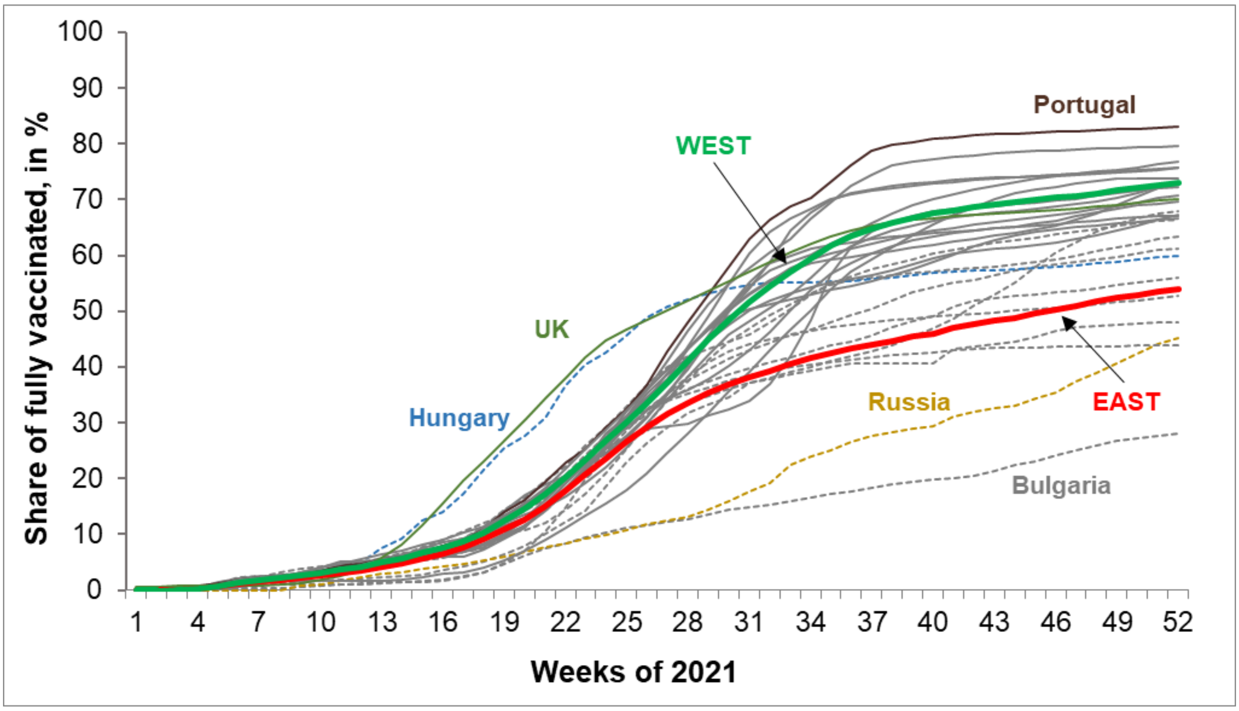

S7 Fig. Cumulative prevalence of full vaccination by weeks of 2021 across countries and country groups East and West.

This figure demonstrates lower vaccination prevalence in Eastern European countries, particularly in Bulgaria and Russia, compared to Western European countries. It should be noted that Hungary is a remarkable exception for higher vaccination prevalence among Eastern European countries.

Data shown in this Figure is provided at https://github.com/VMSdemo/East-West-contrast-in-life-expectancy-losses-in-2020-21
