# Supplementary material for: Widening East-West inequality in life expectancy in Europe during the COVID-19 pandemic: An international comparative study
Source: PLoS One. 2026 Feb 27;21(2):e0344003. doi: 10.1371/journal.pone.0344003 (PMC12948044; doi:10.1371/journal.pone.0344003)
Supplement: S1 Table — (PDF) [file pone.0344003.s015.pdf]

S1 Table. Populations and regional groups under study

East, 11 countries		West, 17 countries	
Country name	Abbreviation	Country name	Abbreviation
Bulgaria	BGR	Austria	AUT
Czechia	CZE	Belgium	BEL
Estonia	EST	Switzerland	CHE
Croatia	HRV	Germany	DEUTNP
Hungary	HUN	Denmark	DNK
Lithuania	LTU	Spain	ESP
Latvia	LVA	Finland	FIN
Poland	POL	France	FRATNP
Russia	RUS	England and Wales	GBRTENW
Slovakia	SVK	Northern Ireland	GBR_NIR
Slovenia	SVN	Scotland	GBR_SCO
		Greece	GRC
		Italy	ITA
		Netherlands	NLD
		Norway	NOR
		Portugal	PRT
		Sweden	SWE

In one of our analyses, we have the UK as a unit of observation with the abbreviation GBRTNP.
