# Supplementary material for: Widening East-West inequality in life expectancy in Europe during the COVID-19 pandemic: An international comparative study
Source: PLoS One. 2026 Feb 27;21(2):e0344003. doi: 10.1371/journal.pone.0344003 (PMC12948044; doi:10.1371/journal.pone.0344003)
Supplement: S2 Table — (PDF) [file pone.0344003.s016.pdf]

S2 Table. Mean number of flights per day to each country for the period 10 February to 23 February 2020

Destination country	Mean number of flights per day	Mean number of flights per day from Eastern Europe	Mean number of flights per day from Western Europe
Eastern Europe			
Bulgaria	3.25	1.00	2.75
Croatia	30.43	2.86	27.57
Czech Republic	110.21	19.93	90.29
Estonia	32.14	8.21	23.93
Hungary	105.86	12.71	93.14
Latvia	73.50	21.93	51.57
Lithuania	6.36	2.57	4.42
Poland	240.86	19.93	220.93
Russia	186.93	37.43	149.50
Slovakia	15.71	4.21	11.50
Slovenia	13.43	2.36	11.07
The mean number of flights per day to Eastern Europe = 816.4 (74.2 per country)			
Western Europe			
Austria	268.86	31.07	237.79
Belgium	228.79	29.36	199.43
Denmark	244.36	26.43	217.93
Finland	121.79	24.86	96.93
France	584.43	48.14	536.29
Germany	1057.93	176.93	881.00
Greece	73.86	7.36	66.50
Italy	639.21	70.71	568.50
Netherlands	340.07	35.29	304.79
Norway	159.43	20.00	139.43
Portugal	258.57	6.43	252.14
Spain	672.79	31.43	641.36
Sweden	224.64	28.71	195.93
Switzerland	394.86	31.43	363.43
United Kingdom	1085.93	126.79	959.14
The mean number of flights per day to Western Europe = 6355.5 (372.7 per country)			
