# Supplementary material for: Widening East-West inequality in life expectancy in Europe during the COVID-19 pandemic: An international comparative study
Source: PLoS One. 2026 Feb 27;21(2):e0344003. doi: 10.1371/journal.pone.0344003 (PMC12948044; doi:10.1371/journal.pone.0344003)
Supplement: S3 Table — (PDF) [file pone.0344003.s017.pdf]

S3 Table. Mean, maximal, and minimal life expectancy (LE) losses (95% CI) in groups West and East in 2020 and 2021, in years

	**Males**	**Females**
	**West: 17 countries**	
	2020	
Mean	0.88 (0.76, 1.00)	0.62 (0.49, 0.76)
Max (m-Italy, f-Spain)	*1.55 (1.40, 1.69)*	*1.36 (1.20, 1.52)*
Min (Norway)	*-0.06 (-0.13, 0.00)*	*-0.07 (-0.14, 0.01)*
	2021	
Mean	0.97 (0.80, 1.16)	0.66 (0.47, 0.86)
Max (Greece)	*1.85 (1.61, 2.08)*	*1.29 (1.01, 1.58)*
Min (m-Norway, f-Sweden)	*0.13 (0.03, 0.23)*	*0.17 (0.04, 0.29)*
	**East, 11 countries**	
	2020	
Mean	1.25 (1.12, 1.38)	0.98 (0.86, 1.11)
Max (Russia)	*2.33 (2.17, 2.50)*	*2.14 (2.03, 2.25)*
Min (Estonia)	*0.49 (0.25, 0.74)*	*0.3 (0.21, 0.39)*
	2021	
Mean	2.89 (2.65, 3.13)	2.52 (2.30, 2.74)
Max (m-Bulgaria, f-Russia)	*3.87 (3.76, 3.98)*	*4.37 (4.21, 4.53)*
Min (Slovenia)	*1.42 (1.17, 1.66)*	*0.90 (0.67, 1.14)*

The table shows that life expectancy losses were higher for males than females. In 2021, the losses were slightly higher than those in 2020 in the West and much higher than those in 2020 in the East. Although the East-West difference in the losses existed already in 2020, it became really drastic in 2021.

Notation

m= male; f= female; Max= Maximum for year and region; Min= Minimum for year and region.

Data shown in this Table (together with the calculations) is provided at https://github.com/VMSdemo/East-West-contrast-in-life-expectancy-losses-in-2020-21 .
