# Supplementary material for: Widening East-West inequality in life expectancy in Europe during the COVID-19 pandemic: An international comparative study
Source: PLoS One. 2026 Feb 27;21(2):e0344003. doi: 10.1371/journal.pone.0344003 (PMC12948044; doi:10.1371/journal.pone.0344003)
Supplement: S4 Table — (PDF) [file pone.0344003.s018.pdf]

S4 Table. Contribution of relative mortality excess and baseline mortality to the East-West difference in life expectancy losses in 2021

	Males	Females
LE losses in the East (Δ_E)	3.02	2.64
LE losses observed West (Δ_W)	0.99	0.67
Differences in LE losses between East and West (Δ_{E-W})	2.03	1.97
LE losses due to larger relative mortality excess (Δ_C)	1.78	1.86
LE losses due to higher baseline mortality (Δ_L)	0.25	0.11
Contribution of the relative mortality excess, %	87.7	94.3

In this Table, life expectancy losses for East and West were calculated in life tables from the average (concerning countries) baseline and observed age-specific death rates. This makes a small difference from Table S3, where losses in the East and the West were calculated by simple averaging of country-specific life expectancy losses. The decomposition results suggest a major contribution of the relative mortality excess (Change component) compared to the baseline mortality (Level component). For example, for males the East-West difference in life expectancy losses in 2021 was 2.03 years (3 years in the East vs. 1 year in the West). This total difference includes 1.78 years (or 87.7%) due to the Change component and 0.25 years (or 12.3%) due to the Level component.

Notation
LE= life expectancy.
Data shown in this Table is provided at https://github.com/VMSdemo/East-West-contrast-in-life-expectancy-losses-in-2020-21
