# Supplementary material for: Widening East-West inequality in life expectancy in Europe during the COVID-19 pandemic: An international comparative study
Source: PLoS One. 2026 Feb 27;21(2):e0344003. doi: 10.1371/journal.pone.0344003 (PMC12948044; doi:10.1371/journal.pone.0344003)
Supplement: Attachment — Submitted filename: Response to Reviewers.docx [file pone.0344003.s020.docx]

**Comments and responses**

| **Reviewer #1** | **Our Responses** |
| --- | --- |
| C0. Overall, the manuscript is well written, with a storyline that is well supported by statistical and demographic analysis. I have a few comments that are listed below. | We are pleased by this positive evaluation. |
| ***General Comments*** | |
| C1. I agree with the factor listed, and I found the flight connectivity a very good indicator despite the limitation listed; however, I couldn’t help to think that for the European countries, trains are also an important source of connection, especially within countries. So, I don’t think it is necessary to add this as an additional factor in the analysis, but a couple of sentences in the discussion will be nice. | We agree. Although the air connectivity is a reasonable proxy for the overall transport connectivity, ground transportation is another substantial but invisible component that is not considered in our analysis.  This is acknowledged now in the revised Discussion (p. 19). |
| C2. In the last paragraph of the conclusions, the author indicated that life expectancy recoveries were larger across Eastern European countries compared to the West after 2021, and that this deserves further investigation. I agree, but, if possible, I would recommend that the authors highlight potential reasons for that. | At the end of the revised Conclusions (p. 20), we indicate two possible factors. Namely, these are a stronger harvesting effect and a higher immunity in the East due to higher mortality and COVID-19 morbidity in 2021. |
| C3. Also, given that is a demographic analysis, I missed a discussion about sex differences, or at least some results by sex. | The male disadvantage in life expectancy losses has been described in earlier studies (references 8 to 10). Throughout our study, all quantitative results are reported for each sex in the text, tables, and figures. The sex difference in the losses was somewhat peripheral in our study since the East-West contrast was large in both sexes.  Nevertheless, we added a paragraph to the revised Results (p. 11). It acknowledges higher LE male losses everywhere and highlights higher female losses in Russia and Bulgaria in 2021 as the only exception. |
| ***Minor Comments*** | |
| C4. Association between life expectancy losses and factors. -I agree with the factors and the conceptual framework. But I would ask the authors to explain each indicator, so, for example, what trust enforcement of policies and regulations means, and how it overlaps with policy on non-pharmacological intervention. | Explanations of the explanatory indicators are given in the Supplementary Data and Methods (p. 5-6). We are sorry that several references to the Supplementary Data and Methods were missing in the original main text. These references are now added to the revised Data and Methods.  Correspondingly, the Materials and Methods section now includes six new references to the Supplementary Data and Methods. The revised Supplementary Data and Methods section now provides more explanation for the two composite indices, the stringency index and the regulatory enforcement score, including their potential relationships to the non-pharmaceutical interventions.  These two composite indices have different senses and are based on different types/sources of data. |
| C5.Association between life expectancy losses and factors. -In the supplementary material, they can describe it in more detail and explain what variables are considered for constructing the indices. Especially to know if there is an overlap in the variables used to construct the indices. | Please note our response to Comment 4.  The variables used to construct the two composite indices do not overlap. Other (single-question) explanatory variables have different senses and do not overlap.  Some statistical associations among them are certainly possible. |
| C6. Association between life expectancy losses and factors.  -Probably is already in one of those indices, but is there an indicator of the labour market, like the share of individuals working from home, especially for 2021? | The workplace closures and distant work requirements are one of the components of the stringency index.  In response to this comment, we have additionally examined the Eurostat’s percentage of employed persons working from home for men and women in 2021 from  https://ec.europa.eu/eurostat/databrowser/view/ lfsa_ehomp__custom_18422919/default/table.  We found that this variable is correlated with life expectancy losses, but it cannot significantly contribute to the explanation of the East-West gap in 2021. |
| C7. Results -Trends in life expectancy in 2000-2023, I would recommend including an additional figure showing the trends in the gap in life expectancy at birth between East and West to have a clear picture of the trends. | Thank you for the comment. This comment refers to Supplementary Figure S2. We have revised this figure by adding an additional panel (S2B Fig) that shows the trends in life expectancy at birth for East and West separately, as well as the gap between them, to provide a clearer picture of the trends. |
| C8. Results Trends and differences in weekly excess mortality.  -I would avoid the word trends, given that you are selecting a specific month, not changes over time. | We agree.  In the revised Materials and Methods (p. 6), the word “trends” is now removed from the subtitle. |
| C9. Results Air connectivity and excess mortality -Can you explain in more detail the rank order correlations? | A transparent explanation of the reasons for and the calculation of the rank correlation coefficients are given in the Supplementary Data and Methods (p. 3-4). We are sorry for not giving a reference to the supplementary in the original text.  The reference is given now on p. 6 of the revised text in the Materials and Methods. |
| C10. Results Figure 4. -I understand that the colour indicates West or East, and the bars are for 0-64 years old and 65+, but I strongly recommend adding a proper legend. | Thank you for the comment. We have now added a proper legend to Figure 4 for clarity. The reviewer understood correctly that the colors indicate West and East, and the bars represent the age groups 0–64 years and 65+. |
| C11. Discussion -Very nice discussion. | We are pleased to see this nice evaluation. Many thanks! |
